# In-hospital cardiac arrest resuscitation performed by the hospital emergency team: A 6-year retrospective register analysis at Danderyd University Hospital, Sweden

**DOI:** 10.12688/f1000research.15373.1

**Published:** 2018-07-06

**Authors:** Hedwig Widestedt, Jasna Giesecke, Pernilla Karlsson, Jan G. Jakobsson

**Affiliations:** 1 Department of Anaesthesia & Intensive Care, Institution for Clinical Sciences, Danderyds University Hospital, Karolinska Institutet, Stockholm, 182 88, Sweden; 2 Clinicum- Centre for Clinical Skills, Interprofessional Education and Advanced Medical Simulation, Danderyds University Hospital, Stockholm, 182 88, Sweden

**Keywords:** Cardiac Arrest, in-hospital resuscitation, CPR, 30-day mortality

## Abstract

**Background: **Cardiac arrest requires rapid and effective handling. Huge efforts have been implemented to improve resuscitation of sudden cardiac arrest patients. Guidelines around the various parts of effective management, the *chain of survival*, are available. The aim of the present retrospective study was to study sudden in-hospital cardiac arrest (IHCA) and the outcomes of emergence team resuscitation in a university hospital in Sweden.

**Methods: **The Swedish Cardiopulmonary Resuscitation Registry was used to access all reported cases of IHCA at Danderyd Hospital from 2012 through 2017. Return of spontaneous circulation (ROSC), discharge alive, 30-day mortality and Cerebral Performance Scales score (CPC) were analysed.

**Results: **574 patients with cardiac arrests were included in the study: 307 patients (54%) had ROSC; 195 patients (34%) were alive to be discharged from hospital; and 191 patients (33%) were still alive at day-30 after cardiac arrest. Witnessed cardiac arrests, VT/VF as initial rhythm and experiencing cardiac arrest in high monitored wards were factors associated with success. However, 53% of patients’ alive at day-30 had a none-shockable rhythm, 16% showed initially a pulseless electrical activity and 37% asystole.

CPC score was available for 188 out of the 195 patients that were alive to be discharged: 96.5% of patients where data was available had a favourable neurological outcome, a CPC-score of 1 or 2 at discharge, and only 6 of these patients had a CPC-score of 3 or higher (3%).

**Conclusions: **One third of patients with sudden IHCA were discharged from hospital and alive at day-30, a clear majority without cognitive deficit related to the cardiac arrest. High monitored care, witnessed cardiac arrest and shockable rhythm were factors associated with high success; however, more than half of surviving patients had initially a none-shockable rhythm.

## Introduction

The importance of prompt recognition of cardiac arrest and initiation of cardio-pulmonary resuscitation has been shown repeatedly ^1, 2^. The chain of survival, prompt recognition, early/bystander cardiopulmonary resuscitation (CPR) and early defibrillation is indeed of outmost importance ^3^. Efforts to improve the results from out-of-hospital have been implemented and our hospital has likewise put training efforts into basic and advanced CPR. 

The aim of the present retrospective register project was to study sudden in-hospital cardiac arrest (IHCA) and the outcomes of emergence team resuscitation at a hospital in Sweden.

## Methods

This is a retrospective single-centre register study in which the Swedish CPR Registry was used to access all reported cases of IHCA in Danderyd Hospital, Stockholm, Sweden. The study protocol was approved by Stockholm Ethical Review Board (EPN; 2017/4:10 approved 2017-11-08, Annika Sandström). Patient informed consent is not required for register studies in accordance with Swedish research regulations.

All reported cases of IHCA at Danderyd Hospital where CPR was initiated, from January 1 ^st^ 2012 to December 31 ^st^ 2017, were included in the study.

Place of cardiac arrest, witnessed cardiac arrest, bystander CPR, time to initiated CPR, initial rhythm, number of defibrillations, patients with return of spontaneous circulation (ROSC), patients discharged from hospital, Cerebral Performance Scales (CPC) score of discharged patients and 30-day mortality was studied.

### Statistics

Data is presented as mean and standard deviation and frequencies as applicable. Differences has been studied by Student’s t-test and ANOVA for continuous variables and Chi sqaured test for category data. A p<0.05 has been considered statistically significant. Statistics has been calculated with SPSS Statistics ^®^ for Macintosh version 24 (Armonk, New York, USA) and Microsoft Excel © 2017 version 16.9.

## Results

A total of 574 patients with sudden IHCA were included in the study: 340 males and 234 females, with a mean age of 73 ± 14 years: 72 ± 13 for males and 75 ± 14 for the females (p<0.05).

The most common place for a sudden cardiac arrest was the Coronary Care Unit (CCU) followed by cardiology and medical wards. A majority (84%) of the cardiac arrests were witnessed, and bystander CPR was initiated within one minute in 96% of cardiac arrest cases. The most common initial rhythm was asystole (n=215) and the least common was VT/VF (n=147). The highest prevalence of VT/VF (57%) was seen in the percutaneous coronary intervention lab followed by the CCU and Intensive Care Unit (ICU) (33% and 27%).

**Figure 1.**
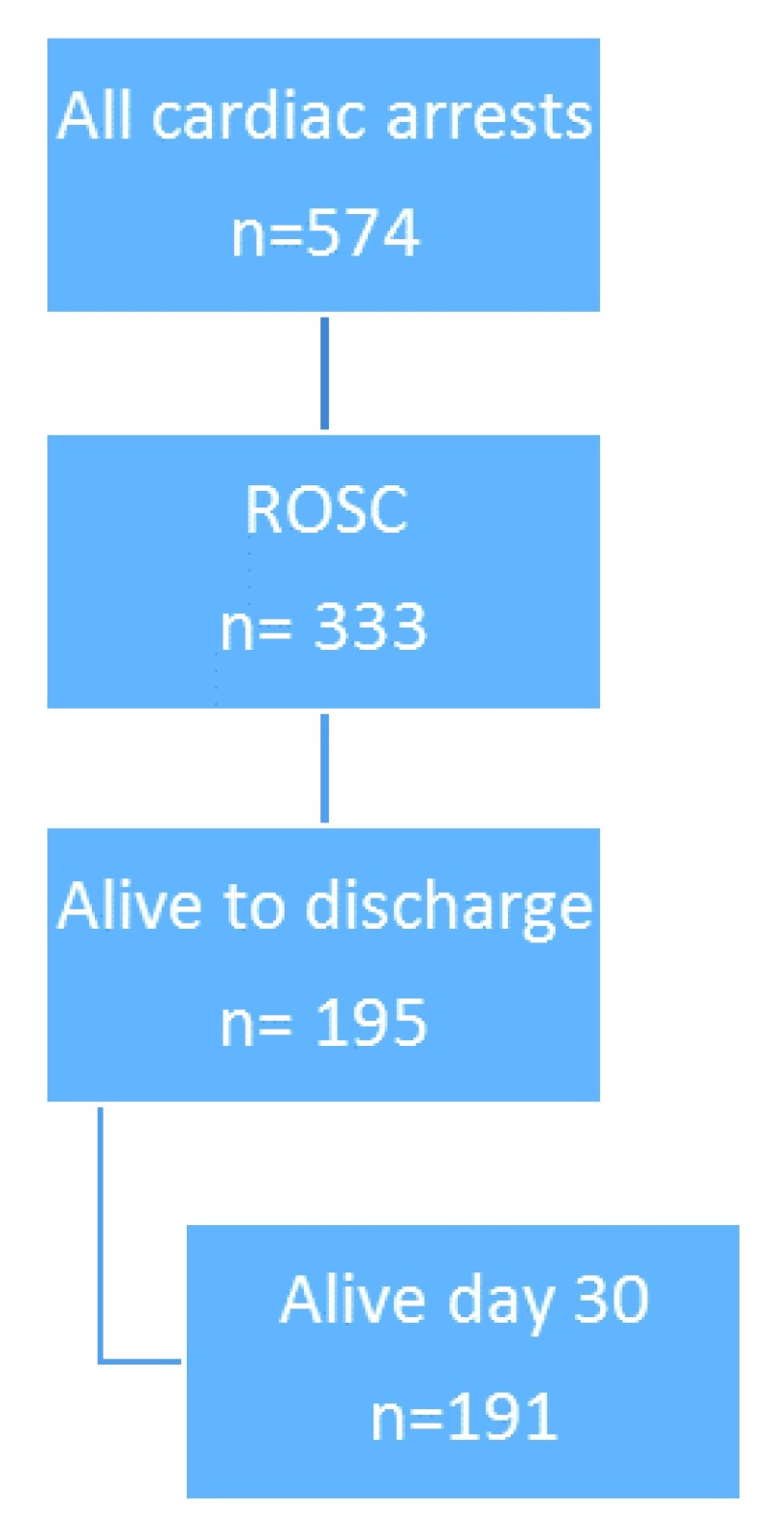
Outcome of resuscitation of in-hospital cardiac arrest. ROSC, return of spontaneous circulation.

In total, 333 (55.5%) of cardiac arrest patients were successfully resuscitated and had ROSC: 195 patients (34%) were discharged from hospital and 191 (33%) were still alive at day-30 after cardiac arrest (33%), see Figure 1.

The highest 30-day survival rate was seen in patients with cardiac arrest in the PCI lab (61%), with the next to highest 30-day survival rate (46%) seen in the CCU.

Shockable rhythm was associated with success: CCU, VT/VF alive at day-30 had 21 out of 28 patients (75%); PCI, VT/VF alive at day-30 had 26 out of 35 patients (74%); and ICU, VT/VF alive at day-30 had 7 out of 12 patients (58%). Overall 89 out of the 167 patients (53%) alive at day-30 had an initial none-shockable rhythm. Age had an impact: patients alive at day-30 were significantly younger than those who were not alive at day-30 (69 *vs* 75 years; p=0.001) ( Table 1).

**Table 1.** Factors with impact on successful outcome of resuscitation of in-hospital cardiac arrest.

	Alive Day-30 (n=191)	Dead Day-30 (n=383)	*All* *(n=574)*
Gender Male/Female	119/72	221/162	*340/234*
Age years	69 * ± 13	75 ± 13	
Bystander CPR Yes/No	188/2	370/12	*558/14*
Time to Bystander CPR; 0’/1’/2’	174 **/12/5	330/34/18	*504/46/23*
Witnessed CA	183/7	299/84	*482/91*
Initial Rhythm, PEA/Asystole/VT/VF	27/62/78 *	146/149/53	*173/211/131*
Place for CA: High monitoring setting/low monitoring setting	119 **/70	145/232	*264/302*

CA cardiac arrest, CPR cardio-pulmonary resuscitation, PEA pulse-less electric activity, VT ventricular tachycardia, VF ventricular fibrillation. P< 0.001 **, p< 0.05 *alive vs- dead day-30

CPC-score was available for 188 out of the 195 patients that were alive to be discharged (96%). In total, 96.5% of patients where data was available had a favourable neurological outcome after cardiac arrest, i.e. a CPC score of 1 or 2 at discharge.

## Discussion

We found that one third of patients suffering sudden IHCA were alive at day-30 and that patients alive to be discharged did not experience significant impairment of cognitive function. A majority of cardiac arrests were witnessed cardiac arrest and CPR had been initiated within 1–2 minutes. Having VT/VF as an initial rhythm and a lower age of the patient increased the chance of survival. However, it is worth noticing that more than half of the surviving patients had a non-shockable initial rhythm.

A previous study at Danderyd Hospital in the late 1980s found only 9 out of 61 IHCA patients were alive to be discharged (15%) ^4^. The survival rate seen in our study is higher than that presented from a study in Ireland on in-hospital resuscitation in 2011, one year before the start of our study, which found a 27% survival rate of discharge ^5^. The average survival rate in our study is also higher than the survival rate reported from a US survey of in-hospital resuscitation including a total of 838,465 patients ^6^. Data analysed from the Nationwide Inpatient Sample databases between 2003 and 2011 showed a 24.7% overall survival to hospital discharge ^6^. A study conducted in Finland between 2009 and 2011, including 279 adult IHCA patients attended by the medical emergency team in a university hospital's general wards, found a 180-day survival rate of 19% ^7^. They commented on the importance of shockable primary rhythm, monitored/witnessed event and low comorbidity score for survival. One should acknowledge that our study covered the period 2012 to 2017 and all hospital wards, including coronary and general intensive care departments. A study from China revealed a low survival rate where only 9.1% of patients were discharged alive ^8^. Our results are however in line with a previous studies from Sweden. Herlitz *et al.* found a 43% survival rate for discharge among cardiac arrest patients suffering cardiac arrest in wards with monitoring facilities, and a 31% survival rate among cardiac arrest patients in general wards. They also found cerebral function to be favourable in most patients ^9, 10^.

Our study does have limitations. We did not study the causes of cardiac arrest. It should be acknowledged that cardiac arrest cases throughout the hospital were included in the study, not only on cardiac arrest in high dependency wards and in patients with heart disease. There is missing data for initial rhythm in about 10% of cases, which means that conclusions concerning prevalence of different cardiac rhythms must be performed with caution. It should also be acknowledged that we do not have data on time to defibrillation.

To conclude, one third of IHCA patients resuscitated by the emergency team could be discharged alive and were still alive at day-30 in our study cohort, a majority without signs of cognitive impairment related to cardiac arrest. Most cardiac arrests were witnessed and CPR had been initiated within minutes. We found initial shockable rhythm VT/VF to be a factor related to successful CPR, which is similar to what has been shown for out-of-hospital CA; however, it should be noted that more than half of survivors had a none-shockable initial rhythm.

## Data availability

The data has been retrieved from the Swedish CPR register ( https://www.hlr.nu/svenska-hlr-registret/). This is a national database, supported by the Swedish and European Resuscitation Councils. The data can be retrieved by request from CPR register ( https://shlrsjh.registercentrum.se/) following Ethical Review board approval on application ( https://www.epn.se/en/start/).
