# Supplementary material for: In-hospital cardiac arrest resuscitation performed by the hospital emergency team: A 6-year retrospective register analysis at Danderyd University Hospital, Sweden
Source: F1000Res. 2018 Jul 6;7:1013. [Version 1] doi: 10.12688/f1000research.15373.1 (PMC6178903; doi:10.12688/f1000research.15373.1)
Supplement: Peer review history

# Peer review: Referee response for version 1

**DOI:** 10.5256/f1000research.16752.r38715

**Published:** 2018-10-08

**Authors:** David A. Pearson

**Affiliations:** 1 Department of Emergency Medicine, Carolinas Medical Center, Charlotte, NC, USA

This retrospective study demonstrates outcomes after in-hospital cardiac arrest as abstracted from a Swedish cardiac arrest registry. This study demonstrates a significant number of non-shockable initial arrest patients with a good neurological outcome, particularly those in PEA. To make this study more robust, it is essential to clarify the code response team, both personnel and process, as well as to ensure consistent definition of cardiac arrest, which in the study was defined as “CPR was initiated”, which needs more clarification (i.e., chest compressions initiated). Finally, there are multiple studies demonstrating similar positive results after in-hospital cardiac arrest. This study represents another study demonstrating the importance of resisting premature prognostication after in-hospital cardiac arrest, regardless of initial rhythm, as many survive with meaningful neurological recovery.

I have read this submission. I believe that I have an appropriate level of expertise to confirm that it is of an acceptable scientific standard.

# Peer review: Referee response for version 1

**DOI:** 10.5256/f1000research.16752.r37733

**Published:** 2018-09-17

**Authors:** Søren Mikkelsen

**Affiliations:** 1 Mobile Emergency Care Unit, Department of Anaesthesiology and Intensive Care Medicine, Odense University Hospital, Odense, Denmark

The aim of the study was to investigate sudden in-hospital cardiac arrests.

 The authors report that one third of the patients that suffer in-hospital cardiac arrest are discharged alive form the hospital.

 The authors describe that the chances of survival increases with shockable rhythm and lower age.  Furthermore, and somewhat surprising, the authors report that more than half of the patients initially had a non-shockable rhythm.

 General comments:

 The study is a single centre study in which the authors report a survival rate following in-hospital cardiac arrest somewhat higher than described in most other studies.

 Between the lines, this reviewer gets the notion that the medical emergency team is responsible for the favourable results. The study is, however, devoid of speculations on why this apparent increase in survival is found at Danderyd Hospital compared with other hospitals. This is somewhat disappointing. There is ample literature available to support discussions on the potential benefits of applying medical emergency teams and I feel that a discussion on caused for survival should have been discussed.

 The authors report that more than half of the patients that survive to discharge are met with an initial non-shockable rhythm. This is an interesting finding and should have been discussed.

 As it stands, the paper is just a reporting on rather favourable outcomes following in-hospital cardiac arrest and is not delving deeper into causes and explanations.

 Specific comments:

 One sentence is rather difficult to comprehend: “The aim of the present retrospective study was to study sudden in-hospital cardiac arrest (IHCA) and the outcomes of emergence team resuscitation in a university hospital in Sweden.”

 Should the sentence read: “The aim of the present retrospective study was to study sudden in-hospital cardiac arrest (IHCA) and the cerebral outcomes following resuscitation by an emergency team in a university hospital in Sweden.”

 Statistics:

 When applying means, standard deviations and t-test, the tested variables should follow a normal distribution. Have the authors assured that?

 References:

 The list of references is rather small and do not support a deeper discussion of results. Not one reference to the concept of medical emergency teams is made.

 Although apparently not the scope of this paper, a more comprehensive discussion of medical emergency teams would have been in order and the list of references should have reflected that.

I have read this submission. I believe that I have an appropriate level of expertise to confirm that it is of an acceptable scientific standard.
